# Measurements and Modelling of Base Station Power Consumption under Real Traffic Loads ^†^

**DOI:** 10.3390/s120404281

**Published:** 2012-03-28

**Authors:** Josip Lorincz, Tonko Garma, Goran Petrovic

**Affiliations:** FESB-Split, University of Split, R. Boskovica 32, Split 21000, Croatia; E-Mails: tonko.garma@fesb.hr (T.G.); goran.petrovic@fesb.hr (G.P.)

**Keywords:** power, energy, base station, measurement, modelling, traffic, consumption, network, wireless, mobile

## Abstract

Base stations represent the main contributor to the energy consumption of a mobile cellular network. Since traffic load in mobile networks significantly varies during a working or weekend day, it is important to quantify the influence of these variations on the base station power consumption. Therefore, this paper investigates changes in the instantaneous power consumption of GSM (Global System for Mobile Communications) and UMTS (Universal Mobile Telecommunications System) base stations according to their respective traffic load. The real data in terms of the power consumption and traffic load have been obtained from continuous measurements performed on a fully operated base station site. Measurements show the existence of a direct relationship between base station traffic load and power consumption. According to this relationship, we develop a linear power consumption model for base stations of both technologies. This paper also gives an overview of the most important concepts which are being proposed to make cellular networks more energy-efficient.

## 1. Introduction

According to [1], approximately 3% or 600 TWh of the worldwide electrical energy is consumed by the information and communication technology (ICT) sector. It is estimated that energy consumption for ICT will grow to 1,700 TWh by 2030. Therefore, it is necessary to find new solutions to reduce the energy consumption of the ICT sector and thus make telecommunication systems “greener”. Since cellular networks constitute a significant part of the ICT sector, reducing consumption of cellular access networks will contribute to the energy consumption reductions of the whole ICT sector.

The growing interest in new and reliable services in mobile telecommunications has resulted in an increased number of installed base stations (BSs) worldwide. In addition, the traditional concept of BS deployment assumes continuous operation in order to guarantee the quality of service anywhere and anytime. Both of these reasons have synergistically contributed during the last decade to the significant growth of the total energy consumed by BSs of cellular network operators. It is well known that the main source of energy consumption in cellular mobile networks is the BS, with a share in total network consumption greater than 50% [2]. Therefore, reducing the energy consumption of BSs as the main energy consumers in the cellular networks has recently become an important research topic.

To reduce the energy consumption of cellular networks, precise knowledge about BS energy consumption and the influence of the traffic load on the instantaneous BS power consumption can be of great importance. Generally, it is assumed that the traffic load variations have small influence on the power consumption of BSs [3,4]. For that reason, this research area lacks papers offering thorough investigation regarding influence of the traffic load variations on the instantaneous power consumption of individual BSs or even complete BS sites. In some recent studies [5,6], authors use a linear models for expressing influence of the traffic load on the instantaneous power consumption of BS. In these studies, linear interdependence was assumed without additional explanation regarding reasons for such assumption.

With the analyses and modelling presented in this paper, we want to offer empirical proof of that assumption, especially for the case of already deployed GSM and UMTS BSs which exploitation time is far from ending. Hence, this paper tackles the issues mentioned by presenting the measurements of power consumption obtained from a real, fully operated BSs site. In addition, according to the measured results, we develop a power consumption model for BSs of different access technologies. The developed model is based on linear interdependence between BS instantaneous power consumption and current traffic load.

The rest of the paper is organized as follows: Section 2 presents an overview of the most promising approaches dedicated to improving the energy-efficiency of the mobile access networks. Descriptions of the BS site at which measurements have been performed with an explanation of the measuring setup are given in Sections 3 and 4, respectively. The results for the power consumption of individual BSs are presented and discussed in Section 5. In addition, Section 6 presents the results of power consumption measurements on the side of the electricity supply grid for the complete BS site. In Section 7, we explain the developed power consumption model for each of the analyzed BSs. Finally, Section 8 gives some concluding remarks.

## 2. Overview of Energy Saving Approaches

Since BSs have the largest share of the energy consumption of cellular networks, it is necessary to identify those elements which contribute most to the overall energy consumption. From the power consumption point of view, the elements of a BS can be divided into two groups: radiofrequency equipment (which includes power amplifiers and transceivers), whose roles are to serve one or more sectors/cells, and support system which includes alternate current/direct current (AC/DC) power conversion modules, air conditioning elements, analogue and digital signal processors, battery backup, *etc.* The largest energy consumer in the BS is the power amplifier, which has a share of around 65% of the total energy consumption [7]. Of the other base station elements, significant energy consumers are: air conditioning (17.5%), digital signal processing (10%) and AC/DC conversion elements (7.5%) [8]. New research aimed at reducing energy consumption in the cellular access networks can be viewed in terms of three levels: component, link and network.

At the component level, investigations are primarily focused on improving the linearity and efficiency of the power amplifier. Efficiency can be improved using a specially designed power amplifier like Doherty, or special materials for power amplifier transistors, like high-frequency materials such as Si, GaAs or GaN. Efficiency can also be improved using techniques such as envelope tracking [9], or by using one of the techniques for crest factor reduction, like peak windowing or amplitude scaling. A digital pre-distortion technique can be used in the power amplifier for cancelling the distortion, and therefore achieving better linearity [10]. The power consumption of the signal processing can be reduced using ASIC, DSP or FPGA architectures of integrated circuits, which are often combined to achieve better efficiency [11]. AC/DC conversion in BSs can be improved using highly efficient converters that can increase their efficiency even in high traffic load situations. Power consumption caused by air conditioning can be reduced by minimizing the operational temperature of base station models, or by using additional elements like heat exchangers, membrane filters and smart fans or heater modules [12].

Additionally, at the component level, energy savings can be achieved by implementing distributed BS architecture, where the radiofrequency equipment is placed near the antennas to minimize the losses in cables [13]. The possibility of installing photovoltaic panels and wind turbines on the base station sites is also being investigated. Even combining these two renewable energy sources can lead to a potential reduction in the power consumption by 50% [14].

The potential for energy savings at the link level is in the transmission techniques on the air interface. Hence, the link level considers possible sleep modes of some BS components (micro and macro sleep), where some of them can be switched off for a certain time. In that case, the BS must provide a certain differentiation between transmissions by scheduling traffic load in the uplink and downlink [8]. The energy efficiency of the BS provided by sleep modes can be increased through implementation of cell wilting and blossoming techniques. These techniques, used for the design of BS sleep and wake-up transients, consist of a progressive BS switching off and on. It is shown that these transients are very short, which allows BSs to be switched off and on in a short time with no significant reduction in the energy savings obtained through sleep mode approaches [15]. The 4G systems are considering the possibility of dynamic allocation of the frequency spectrum depending on traffic load [3]. The cancellation of the interference in cellular networks using distributed antenna systems and algorithms, such as linear zero forcing, minimum squared error and successive interference cancellation, also contributes to the reduction of energy consumption [16].

At the network level, one of the most important approaches for reducing energy consumption is dynamic management of network resources, which allows shutting down of entire BSs during a low traffic load. In such a scenario, neighbouring BSs must provide coverage and take over the traffic load of those BSs that are turned off [8]. This can be combined with dynamic Tx power selection, antenna tilting, multihop relaying or by coordinated multipoint transmission and reception [4].

An important concept for reducing power consumption of the cellular networks is presented in the Energy Efficiency Evaluation Framework (E^3^F), which includes several models that can improve energy efficiency at low traffic load [16]. Models are divided into small-scale short-term and large-scale long-term models. Small-scale short-term models are power models that map RF output power radiated at the BS antenna to the total power supply of the BS site. Large-scale long-term models include traffic models that describe traffic load fluctuations over a day and deployment models that extend existing small-scale deployment models to large geographical areas.

Energy efficiency can also be improved without turning off BSs, using a technique called dimming cellular networks. This technique is constructed as a multi-period optimization problem enabling frequency dimming which switches off certain BS frequency channels. Frequency dimming can then be combined with service dimming, which disables certain high data rate services on enabled frequencies during periods when traffic demand is low [17].

In the case of heterogeneous cellular network architecture, the network itself represents the potential for reducing energy consumption. In this type of cellular network, macro cells are complemented with low transmit power cells like micro, pico and femto cells [3]. Macro cells ensure permanent coverage, while the turning on and off of smaller cells depends on current traffic load. The possibility of applying techniques such as cell zooming, where the cell can adjust its size according to traffic load situation, is also explored in [18]. The potential to reduce the power consumption of cellular networks is also in its planning and operation. One of the proposed models is the Traffic-Aware Network Planning and Green Operation (TANGO) framework, future implementation of which can increase the energy efficiency of cellular networks while keeping QoS at a satisfactory level [19].

Furthermore, some initiatives are based on the possibility of making energy savings through cooperation between competing operators who provide services in the same area of coverage (usually in cities). The point is that one of the operators can completely switch off its BS during low traffic load, while the BS of the second operator accepts the subscribers of the both operators. According to the authors of [20], such an approach can offer reductions in energy consumption by 20%. In [21], the authors propose several solutions that can improve energy efficiency of 4G BSs. These solutions can be observed from time, frequency and spatial domains, with the most promising solutions being hybrid solutions that combine solutions in different domains to adapt the power consumption of BS site to different traffic conditions. Actually, the concurrent use of most approaches mentioned will have a synergistic effect that leads to completely energy-efficient mobile networks of the future.

## 3. Site Description

In order to show the interdependence between BS energy consumption and traffic load, extensive on-site measurements were performed at a fully operated BS site located in an urban-dense area of a medium sized city. The selected BS site is one of the most loaded city sites in terms of voice and data traffic flows. Information on the name of the mobile operator, BS site location, model and manufacturer of the BSs will not be provided, in order to guarantee operator confidentiality. BSs of the four different cellular access technologies: GSM 900, GSM 1800, UMTS and LTE (Long Term Evolution) were located at the measuring site. The LTE BS is currently used by an operator only for the purpose of testing new LTE technology. Since the LTE BS does not offer a 4G service to users and transfers negligible amounts of traffic, it will be excluded from further analysis. Each BS cabinet is an indoor type of cabinet located inside a protected room dedicated solely for keeping site equipment. Antenna lines connect each BS cabinet with corresponding antennas located on top of the building. The site is connected with a backbone network using optical lines over a gigabit switch. An overview of the BS properties installed at the analyzed BS site is shown in Table 1.

The AC power grid to which the BS site is connected has been based on a three-phase low-voltage system (400/230 V), using a TN-S grounding scheme. The non-direct touch protecting system is based on a residual current device characterized with the nominal current of 40 A and differential current of 500 mA. For proper functioning of each BS cabinet, the declared voltage values of direct current (DC) power supply range from 43 V to 56 V. Additionally, the site contains a redundant DC battery supply of 48 V connected by means of a buffer coupling with the site's AC/DC electricity converter. In the most common mode of site operation, characterized by uninterrupted energy supply from the electricity grid, an AC/DC converter gives time invariant DC voltage equal to 53.6 V. Assuming fully charged batteries (checked by measurements), negligible attenuation in cables (proper material, length and diameter) and negligible influence generated by the measuring system itself (current clamps enable non-galvanic bond), this voltage is equal to the voltage on the input of the BS racks. Also, the complete site is air-conditioned and site cooling consumes approximately 1 kWh.

**Figure 1.**
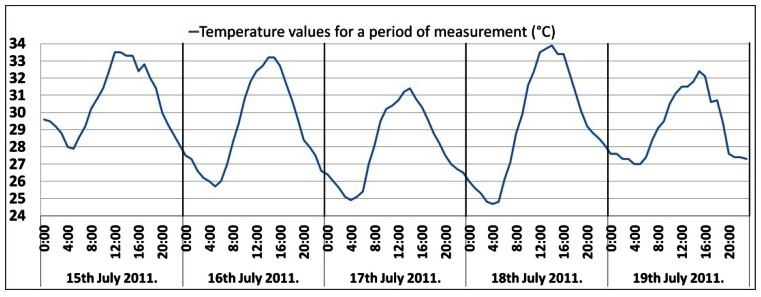
Temperature values for the measurement period.

Furthermore, it is necessary to emphasize the temperature characteristics of the analyzed BS site located in a Mediterranean climate region. Figure 1 shows the daily temperature variations during the measuring period, which are typical for that time of year. According to Figure 1, there was no significant oscillation in the temperature during the measuring period. Hence, the oscillations did not significantly affect the measurements, and due to the presence of the cooling device, their influence on the measurements can be neglected.

## 4. Measuring Setup

**Figure 2.**
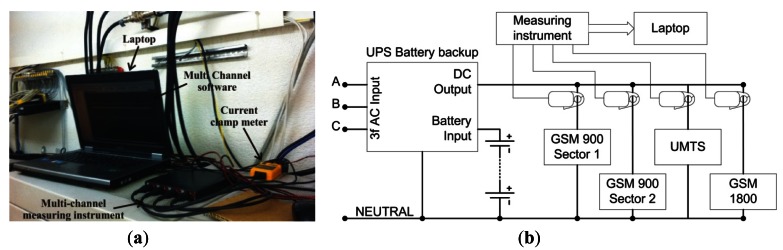
(**a**) Snapshot and (**b**) block diagram of measurement setup used for measuring instantaneous DC electric current consumption of BSs.

In order to measure the electric current drawn on the DC side of the individual BSs installed on the site, the following equipment was used: laptop with specialized measuring software, multi-channel measuring instrument, current clamps and corresponding cables. Snapshot and block diagram of the DC measuring setup are presented in Figure 2. The multi-channel instrument used is the Handyscope HS4 (TiePie Engineering, Sneek, Netherlands). This instrument has the capability of mapping up to four input measuring signals. In our case, measuring signals were generated by Fluke i30s current clamps. In order to accomplish very long continues measurements, we are forced to extend battery life of the current clamps. We perform this through adaptations which enable power supply of the clamps from external DC battery having enough capacity for such measurement. Clamps were used for precise detection of the DC current flowing through the electric supply cable of the BS cabinets. The measured signals are concurrently transferred through a USB cable to the laptop on which the MultiChannel software of the corresponding multi-channel instrument has been installed. This software enables graphical presentation of the measurements with many processing options.

To measure the AC energy consumption of the overall site, including the cooling system (air-conditioning) and battery supply systems, the following equipment components were used: laptop with specialized measuring software Dran-View 6, a three-phase PowerVisa power quality analyzer equipped with 8 independent channels and data-logger, flexible Rogowski current-clamps equipped with the embedded signal integrator for currents of up to 3 kA, voltage terminals, current clamps for measuring currents of up to 10 A, UNI-T True Root Mean Square (TRMS) multimeter and UNI-T non-contact voltage detector. The measured signals were concurrently stored to an embedded 2 GB SD memory card and transferred to the laptop via a corresponding adapter.

**Figure 3.**
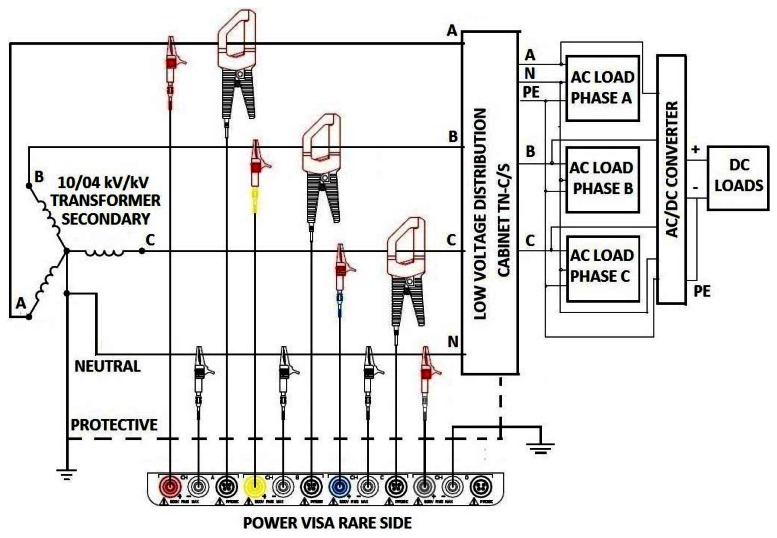
Contacting scheme for measuring AC consumption of compleate BSs site.

The measuring scheme according to which measurements of the power consumption on the AC side of the BS site were performed can be seen in Figure 3. As presented in Figure 3, the voltage channels were connected to phase voltages, while phase currents were measured using current clamps. Since in-situ measurements showed higher current consumption in the phase A (non-symmetric load), the neutral line current was expected to be non-zero and an additional current clamp was placed on the neutral line. In addition, from in-situ measurements it was found that overall site power consumption is dominated by the BS racks as well as the air-conditioning system. Site lighting is based on neon-bulbs normally kept off, except during rare maintenance activities. Therefore, site lighting including safe light consumption can be treated as negligible.

In our research, we take into consideration two GSM 900 BS cabinets dedicated for transferring traffic of Sectors 1 and 2. The residual GSM 900 BS cabinet covering Sector 3 was not analyzed due to significantly smaller average traffic load. In addition, measurements were performed for the GSM 1800 and UMTS BSs, having in a single cabinet equipment for covering all three sectors (Table 1). During the measuring period, we measured in parallel the current draw of four BS cabinets (GSM 900 Sectors 1 and 2, GSM 1800 and UMTS) on each of the four channels of the multi-channel instrument.

For measuring the DC consumption, the frequency of measuring samples was set up to 5 kHz on each of the measuring channels. Furthermore, the samples were filtered using a low-pass filter of 1 Hz and finally re-sampled to 10 Hz, which results in 1 measuring sample every 10 s. Due to slow changes of the BS power consumption during the day, it is reasonable to believe that such an approach guarantees acceptable measurement accuracy. In the case of AC consumption by the overall BS site, sampling was set to 256 samples per cycle (20 ms) and integrated over 1 second intervals.

Continuous five-day measurements were performed on AC and DC side in the period from 15 July starting at 12:00 until 19 July ending at 10:00. It is worth emphasizing that the measuring period starts on Friday and finishes on Tuesday. We selected this measuring period in order to identify possible differences in the BSs power consumption between working and weekend days.

## 5. Measurements of DC Consumption

### 5.1. Instantaneous DC Power Consumption

**Figure 4.**
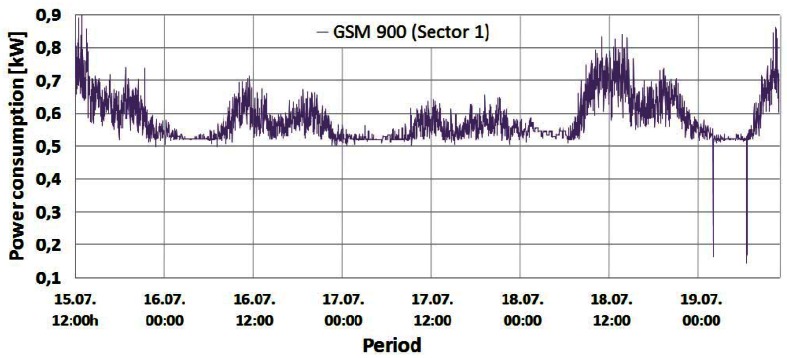
Power consumption of GSM 900 (Sector 1) BS cabinet.

**Figure 5.**
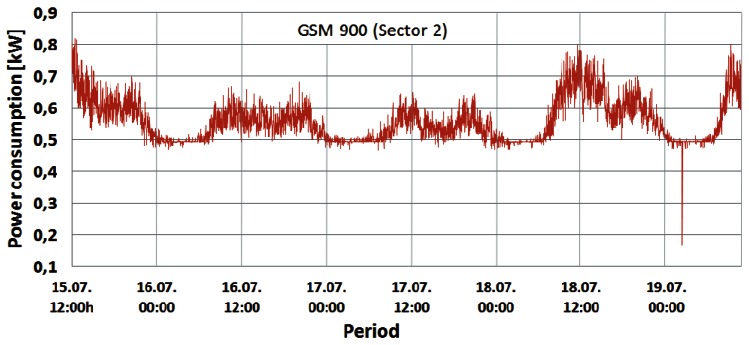
Power consumption of GSM 900 (Sector 2) BS cabinet.

**Figure 6.**
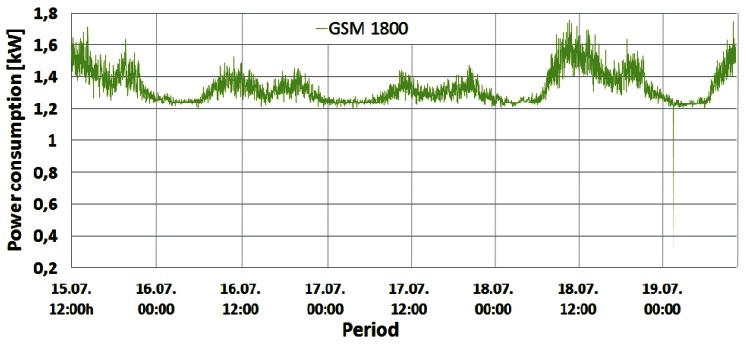
Power consumption of GSM 1800 BS cabinet.

**Figure 7.**
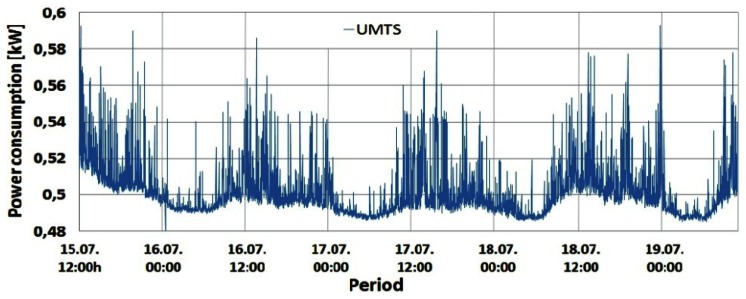
Power consumption of UMTS BS cabinet.

Changes in the instantaneous power consumption of the GSM 900 (Sector 1 and 2), GSM 1800 and UMTS BSs are presented in Figures 4–7, respectively. These power consumptions of the individual BSs were obtained by multiplying the measured values of the instantaneous BS electric current consumption with constant DC voltage (53.6 V). A very short but rapid decline of the power consumption in the early morning hours on the last day of measurements can be perceived in Figures 4–6. Due to extremely short duration, this unexpected decline caused by brief instability of some BS components will not influence the overall measurements and can be neglected. According to Figures 4–7, it is obvious that the power consumption of each BS is not constant in time. Actually, the instantaneous power consumption of the BSs varies during a day and these variations are inherent for all the analyzed mobile technologies (GSM 900, GSM 1800 and UMTS).

From Figures 4–7 it can be seen that the highest power consumer is the GSM 1800 BS. Compared with the BS consumption of the other technologies, this BS has more than twice higher instantaneous power consumption at any moment. This is because the GSM 1800 cabinet serves all three sectors concurrently through configuration with 4 transceivers (TRXs) per sector (4/4/4). Since each TRX has a separate power amplifier, and in Section 2 we show that the power amplifier has the highest share in the BS power consumption, the number of TRXs has an important influence on the total BS power consumption.

Therefore, compared with the number of TRXs in the GSM 900 (7/sector) and UMTS (1/1/1) cabinets (Table 1), the higher total number of TRXs (12) in the GSM 1800 BS cabinet is the main reason for having the highest power consumption in this BS. In addition, the influence on the power consumption will be somewhat determined by the fact that the GSM 1800 BS has been selected for maximum capacity utilization. This means that the GSM 1800 BS accepts voice and text messaging communication first. The other three GSM 900 BSs serve as redundant BSs if the GSM 1800 is fully loaded or when the received signal strength drops below a predefined threshold. Nevertheless, the GSM 900 BSs execute call setup establishment and start of the conversation, which preserves the high activity level of the BSs. From Figures 4 and 5 it can be seen that Sectors 1 and 2 of the GSM 900 BS have a similar power consumption pattern. The BS serving Sector 1 has somewhat higher power consumption during every moment of the day due to higher traffic activity of the users located inside Sector 1. Of all the on-site BSs, the UMTS BS has the lowest power consumption. This can be explained by the configuration having the minimum number of TRXs (1/1/1) and the newer technology characterized with BS hardware which is generally more energy-efficient.

Confirmation of this can be found in Table 2, which gives the minimum and maximum daily power consumptions with the percentile ratio between them for weekend and working days.

Based on Table 2, the lowest oscillations in the daily power consumption were recorded for the UMTS technology (∼20%) while the highest variations were obtained for the GSM 900 BSs (34%–43%). Although the GSM 900 and 1800 BSs are of the same technology and manufacturer, the GSM 1800 BS with an even higher number of TRXs has lower variations in daily power consumption (22%–32%). This is a direct result of hardware improvements built into the newer GSM 1800 and UMTS BSs manufactured in 2009 and 2010 (Table 1), respectively. In some recent analyses dedicated to improving the energy-efficiency of the cellular networks, these variations are neglected, assuming constant power consumption of BSs. This assumption is obviously incorrect, but it ensures significant simplification when expressing BS power consumption. On the other hand, such simplification can lead to wrong estimation of BSs' monthly energy consumption. This is because daily energy consumption of BSs is somewhat different for different days of the week (Table 2). Generally, higher energy is consumed during the working day (Monday) and small differences in the energy consumption can even be seen between weekend days.

### 5.2. Interdependence of Current Draw and Traffic Load

The shape of the power consumption pattern presented in Figures 4–7 is basically identical for all BSs and does not depend on the transmission technology. This means that the highest power consumption was recorded between 10:00 and 14:00 (peak hours) for each of the analyzed days. In addition, increased consumption was recorded in the period from 19:00 to 22:00, while the lowest consumption was observed between 02:00 and 07:00. Also, during peak hours, for each BS differences in the power consumption between working (15, 18 and 19 July) and weekend (16 and 17 July) days can be clearly perceived. Figures 4–7 show that BSs during the peak hours of the working days have higher power consumption compared with consumption during the peak hours of the weekend days (Table 2).

**Figure 8.**
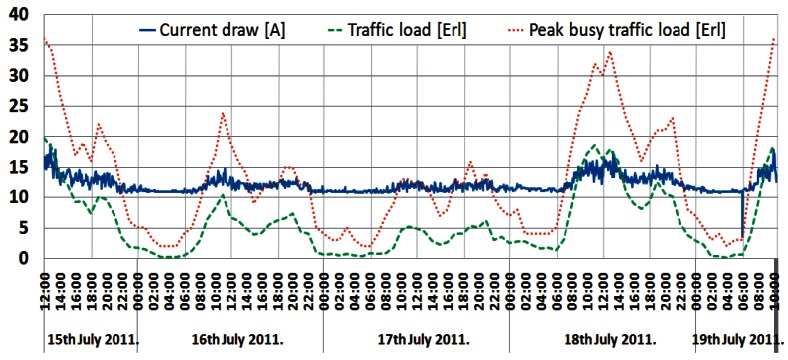
Comparison between electric current draw and traffic load for GSM BS (Sector 1).

**Figure 9.**
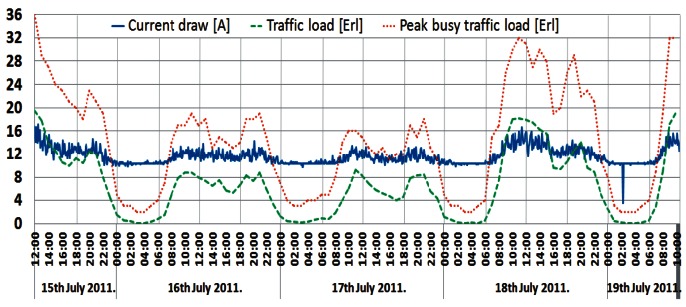
Comparison between electric current draw and traffic load for GSM BS (Sector 2).

**Figure 10.**
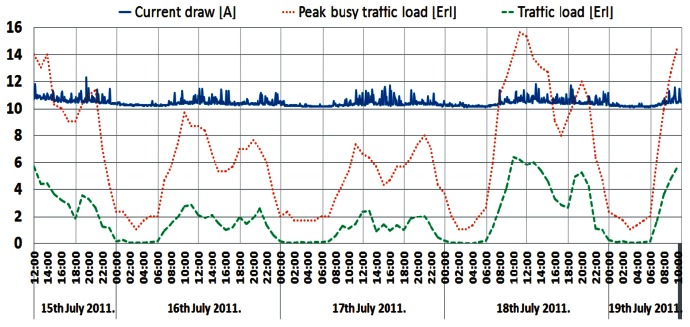
Comparison between electric current draw and traffic load for UMTS BS.

The shape of this power consumption pattern is a direct consequence of a daily traffic pattern variation. It can be said that the increase in user activity during a day results in an increase in the instantaneous power consumption of a BS and vice versa. Confirmation of this can be found in Figures 8–10, presenting a comparison between measured traffic load and electric current draw for the GSM 900 Sector 1, Sector 2 and UMTS BSs, respectively. Information regarding average traffic load and peak (maximal) traffic load recorded on an hourly basis was obtained from the specialized monitoring system of an operator. In the case of the UMTS BS traffic load, it is worth emphasizing that we present in Figure 10 the averaged traffic load of all three sectors.

Graphs presented in Figures 8–10 show that changes in the instantaneous electric current closely follow variations of the peak and average traffic loads. Hence, direct correlation between the electric current draw and variations in the traffic pattern can be perceived. A somewhat lower level of correlation can be noticed in Figure 10 for the UMTS current draw, due to the minimum number of TRXs in the BS configuration (1/1/1). In addition to this, only the one-year-old UMTS BS has newer hardware which is less prone to traffic variations. Although the level of correlation in graphs in Figures 8–10 might seem negligible, it is a consequence of the presentation approach, which uses the same axes to present different measuring parameters (Erlangs and Amperes). On the other hand, the influence of the correlation is highly reflected in the power consumption (Figures 4–7), which explains its non-negligible daily variations.

In terms of generating voice or data traffic, user activity during the first quarter of a day is low and the current draw of the BSs will be lower. On the other hand, during peak hours, user activity is high and BSs' consumption of the electric current becomes higher. The explanation of such behaviour can be found in the additional hardware and processing resources that must be activated by a BS in order to accommodate increased traffic load. Therefore, typical day/night variations in the user's activity have an influence on the BSs' power consumption.

### 5.3. Role of Transceivers in BS Power Consumption

Additional measurements were performed on the DC side of the BS site in order to deduce the influence of the individual TRXs' consumption on overall BS power consumption. The TRX is the most energy-demanding part of the BS hardware and in Section 5.1 we show that number of TRXs inside the BS cabinet has an influence on the overall power consumption. To show the impact of the number of TRXs on BS power consumption, we performed simultaneous measurements of current draw on three BSs: GSM 900 (Sector 1), GSM 1800 and UMTS. After every two minutes of continuous measurement, we separately turned off one by one the TRXs of the GSM 900 and 1800 BSs until all the TRXs were switched off. Measurements were done during the peak hours (10:56–11:45) of a working day, since results presented in Figures 4–10 show the highest influence of the traffic load on the power consumption during that period.

**Figure 11.**
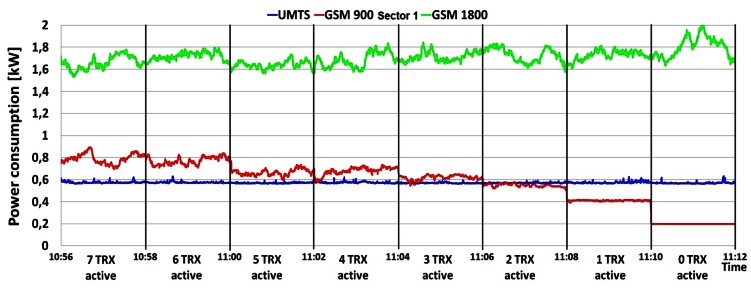
Power consumption of BSs during deactivation of TRXs in GSM 900 BS cabinet.

**Figure 12.**
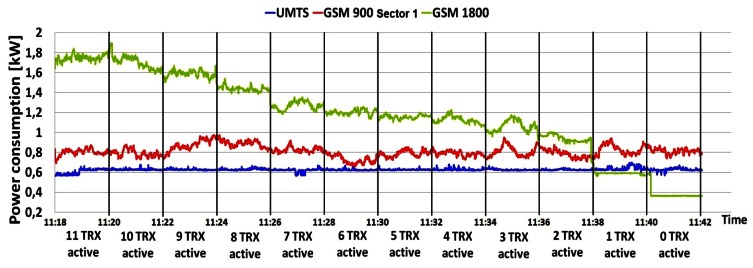
Power consumption of BSs during deactivation of TRXs in GSM 1800 BS cabinet.

Figure 11 presents the measurements obtained in the case when TRXs of the GSM 900 (Sector 1) BS were sequentially deactivated. As expected, instantaneous power consumption by the GSM 900 BS decreases after turning off each consecutive TRX by approximately 50 W. Similar behaviour of the instantaneous power consumption with a somewhat faster decrease trend (approx. 100 W decrease steps) can be observed in Figure 12 when TRXs of the GSM 1800 BS were deactivated. Higher decrease steps for the case of the GSM 1800 BS are due to the higher traffic load served by this BS and different TRX technology.

On the other hand, Figure 11 shows that the decrease in the power consumption of the GSM 900 BS was followed by an increase in the power consumption of the GSM 1800 BS. This is because the GSM

1800 BS starts to accept an increased traffic load coming from the turned off TRXs of the GSM 900 BS. A reversal of this behaviour in the power consumption changes can be seen in Figure 12, with the accent on the constant power consumption of the GSM 900 BS at maximal levels. In this situation, the GSM 900 BS is forced to work permanently with maximum TRX configuration due to the necessity of accepting traffic which would otherwise be served by the GSM 1800 BS. There is no significant increase in the power consumption of the GSM 900 (Sector 1) BS due to the fact that the other two GSM 900 BSs (Sectors 2 and 3) also partially accept traffic from the turned off TRXs of the GSM 1800 BS.

In addition, it can be noticed from Figure 12 that the almost constant power consumption of the UMTS BS starts to increase at the moment when the GSM 1800 BS is left with one or no active TRX. At that moment, the UMTS BS primarily dedicated for data transfer starts to accept voice communication, which leads to an increase in the power consumption. This increase cannot be seen in Figure 11 since the GSM 1800 BS has enough capacity to accommodate traffic coming from the switched-off transceivers of the single GSM 900 BS covering a single sector. Hence, there is no need to accept additional voice traffic by the UMTS BS and consequently there is no increase in the BS power consumption. Trends describing the behaviour of BSs' power consumption presented in Figures 11 and 12 additionally confirm the impact of the traffic load on the BS power consumption.

Sequential shutting down of TRXs inside the UMTS BS was not performed due to operator restrictions emphasizing the minimal BS configuration (1/1/1) which can lead to complete loss of the UMTS service. Nevertheless, Figures 11 and 12 reveal that completely shutting down of all TRXs inside the GSM 900 or 1800 BS will not result in loss of the voice or data service offered by the analyzed BS site. This is in compliance with common network planning principles, according to which locations with higher traffic load must be covered with redundant capacity. Furthermore, the graphs in Figures 11 and 12 show that even when all the TRXs on each analyzed BS were turned off, the BSs still consumed some constant power. This consumption is generated by the other BS hardware components like the AC/DC adapters, cooling fans, main processor, *etc.*

## 6. Measurements of AC Consumption

### 6.1. Active Site Power Consumption

**Figure 13.**
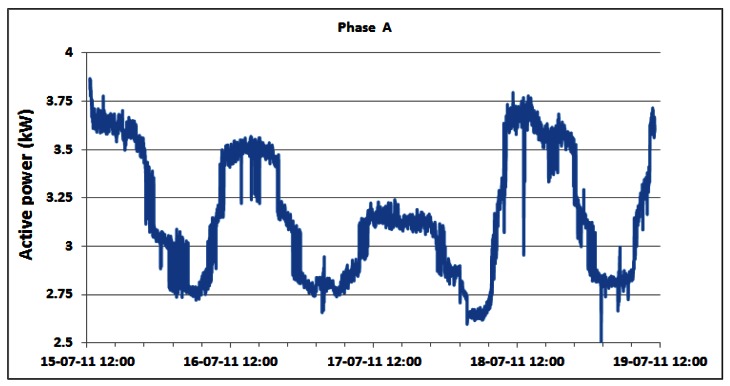
Active power of phase A for15–19 July.

**Figure 14.**
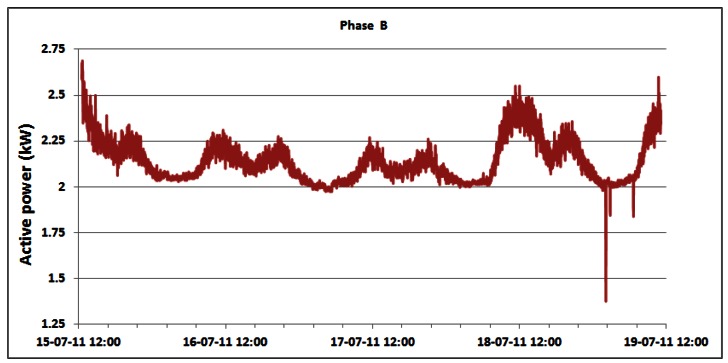
Active power of phase B for 15–19 July.

**Figure 15.**
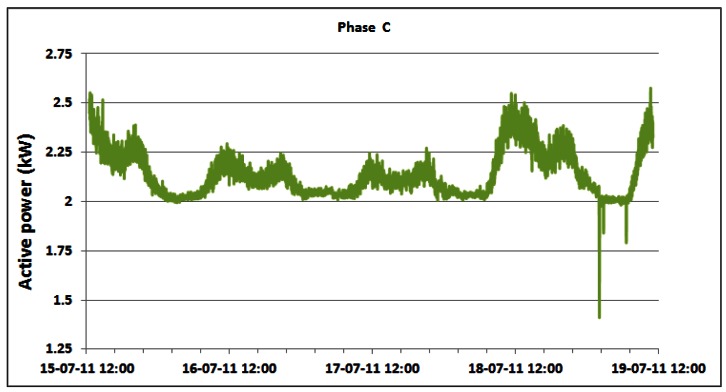
Active power of phase C for 15–19 July.

Contrary to the power consumption measurements on the DC side, the AC power consumption of the overall BS site is measured directly using the setup described in Section 4. Changes in the AC power consumption of the overall BS site for phases A, B and C of the three phase electric supply system are shown in Figures 13–15, respectively.

**Figure 16.**
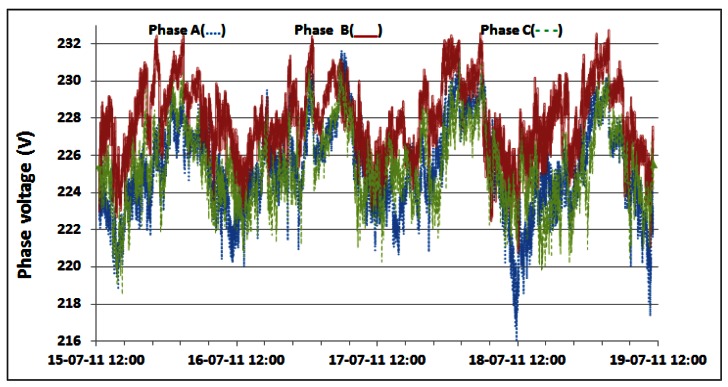
Phase voltage oscillations for 15–19 July.

As a direct consequence, variations in the measured active power are primarily caused by the previously presented variations in the power consumption of the individual BSs. Small variations of phase voltages presented in Figure 16 give negligible contribution to variations of active power consumption. In addition, somewhat more visible variations are in phase A where oscillations are generated by the air-conditioning system connected in that phase. In Table 3, we present statistics of the active AC power consumption for each day during which continuous measurements were performed. Applying the detailed analyses presented in Table 3, in Table 4 we present results for the average active power consumption of the complete BS site for each of the phases. According to Table 4, average AC active power of the complete BS site during continuous five day measurements is: 3.16 kW, 2.14 kW and 2.14 kW for phases A, B and C, respectively. As can be seen from the calculated averages presented in Table 4, the average consumption for phases B and C is equal.

A discrepancy of approximately 1 kW in magnitude observed in phase A is caused by the single-phase air-conditioning system connected in that phase. Differences between the minimum and maximum average active powers are: 1.39 kW (35.92%), 1.11 kW (48.7%) and 1.16 kW (45.35%) for phases A, B and C, respectively. Analogously to the behaviour of the Min./Max. active power ratio, the Min./Aver. deviations in phases B and C are also quite equal, while the difference in phase A arises from operation of the already mentioned air-conditioning system.

Generally, trends in changes of the AC power consumption pattern during the weekend days (Figures 13–15) are similar to those of the working days. We would like to point out that the first and last day displayed in Table 3 (and Table 2) do not encompass the entire 24 h period, since measurements started on Friday (July 15) at 12:30 and finished on Tuesday (July 19) at 11:06. However, from graphs in Figures 13–15 and Table 3, we can see the following regarding the active energy consumption of BS site: generally weekend consumption is lower than working day consumption. This conclusion arises from comparison of Saturday or Sunday consumption with Monday consumption and illustrates the structure of the mobile telephony use. Reduced consumption during the weekend suggests that most of the cell phone users are workers (active population) and as a consequence, they use the mobile phone less frequently during the weekend.

In order to focus on the daily consumption, active power is analyzed for the individual phases within the 24 h period in Table 3. These results do not significantly differ from the average active power within the total five day period (Table 4). As an example, we will comment on the statistics for Monday (July 18). Observed average values are 3.23 kW, 2.18 kW and 2.19 kW for phases A, B and C, respectively. As has already been pointed out in the analysis of the total active power consumption, the approximately 1 kW higher consumption in phase A is caused by the air-conditioning system. Maximum active AC power consumption on Monday for phase A equals 3.79 kW and for phases B and C is 2.55 kW. These power consumptions were recorded simultaneously in each phase at 11:20. The minimum active power had a magnitude of 2.60 kW for phase A and 2.00 kW for phases B and C.

These values were also detected simultaneously at 02:17.

The lowest and highest values of the active power consumption in each phase were obtained during the same periods of the day as for the case of the instantaneous DC power consumption of individual BSs discussed in Section 5.2. Since traffic variations influence the instantaneous power consumption of the BSs on the DC side, this influence is reflected in the active power consumption in each phase on the AC side of the complete BS site. Therefore, correlation between telecommunication traffic and active AC power consumption in each phase of the three phase system also exists. Reasons for this are quite obvious and arise from the typical day-night behaviour of the users, having different habits regarding time and frequency of cell phone usage during a day.

### 6.2. Distribution of Site Power Consumption

**Figure 17.**
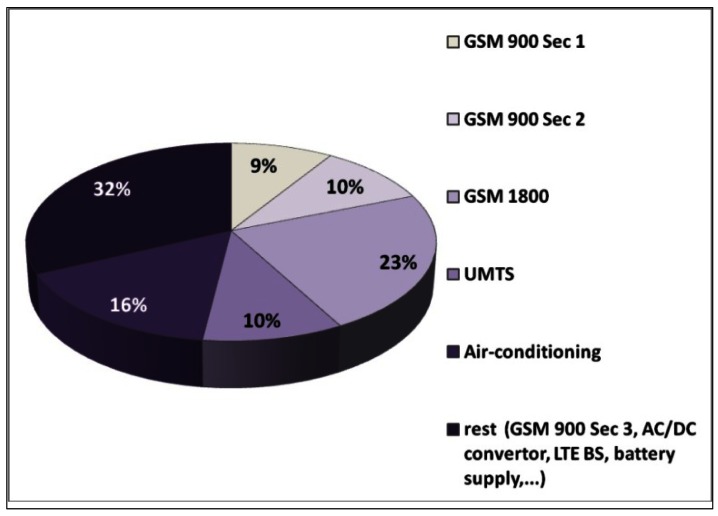
Distribution of the total active power consumption of the analyzed BS site.

Aiming for a better understanding of the active power consumption of the complete BS site, we examine distribution of the active site power consumption among the different equipments installed on the analysed macro BS site. Figure 17 shows the percentage of the active power consumption in the site's total AC power consumption, for each of the analyzed equipments. According to Figure 17, a major fraction (52% cumulatively) of the total site consumption is caused by the analyzed telecommunication equipment, namely the GSM 900 sector 1 and 2, GSM 1800 and UMTS BSs. In addition, if we take into consideration other BSs installed on site (GSM 900 sector 3, tentative LTE), this percentage reaches values of up to 80%. A significant part of the overall consumption, around 16%, originates from the air-conditioning system, while a small percentage of the total site consumption is attributed to the AC/DC converter and battery supply system.

Therefore, reduction in the energy consumption of individual BSs will make a significant contribution to the energy consumption reduction of the complete macro BS site on the AC side. Also, reduced energy consumption of BSs will result in impaired heat dissipation, which consequently results in lower energy consumed for site cooling. Moreover, the appropriate design of macro BS sites, especially in the case of outdoor BS sites with natural air-cooling BS racks, can be used in order to completely eliminate the need for electrically supplied air-conditioning.

Table 5 presents an estimation of the energy consumption and financial costs for the case of the analyzed BS site and overall cellular access network of an operator. The LT, HT and Total in Table 5 stand for Low Tariff energy, High Tariff energy and Total energy, respectively, while the Cost column represents the overall energy price in Euros (€). The calculation is based on the assumption of a dual tariff scheme typical for the European energy markets. The exact €/kWh ratio for each tariff is selected according to the actual electricity prices for the European country in which the BS site is located.

Furthermore, we consider Monday to be representative of working days, while Saturdays and Sundays are treated separately due to the aforementioned differences in the energy consumption. Therefore, estimations were made according to the obtained measurements for Monday, Saturday and Sunday. During one month, we assume for simplicity 22 equal working days represented by the Monday consumption and four weekends represented by the Saturday and Sunday consumption. Therefore, in the case of one month having 30 days, the total energy consumption of the analyzed BS site is 5,347.6 kW. Assuming for simplicity equal energy consumption for each month during a year, total yearly energy consumption of this BS site is 64,171.2 kW. The operator has approximately 2,000 installed BS sites and average energy consumption per site is approximately 60% of monthly/yearly consumption of the analyzed BS site. This is because the site belongs to the group of the largest sites in terms of the number of installed equipment and corresponding energy consumption. Such a conservative approach leads us to a rough esimation of the operator's total yearly energy consumption. This consumption is vast, and on the level of the operator's radio access part of the network, equals approximately 7,700.54 MW. Translated into financial costs, this corresponds to the amazing amount of approximately 5.3 million euros that the operator pays to the electricity supply company.

### 6.3. Reactive Site Power Consumption

**Figure 18.**
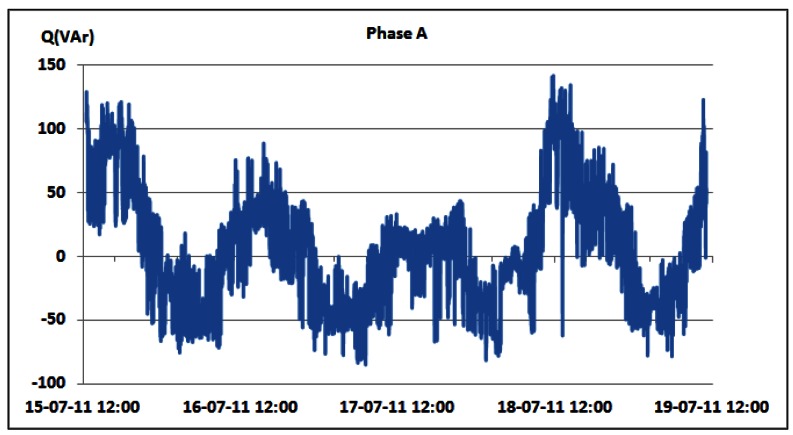
Reactive power of phase A for 15–19 July.

**Figure 19.**
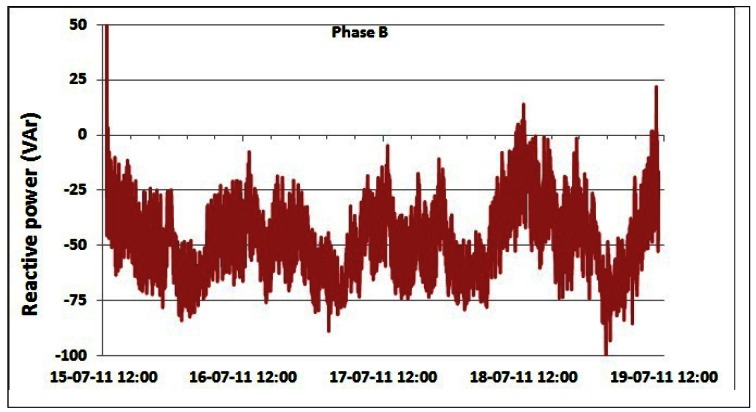
Reactive power of phase B for 15–19 July.

**Figure 20.**
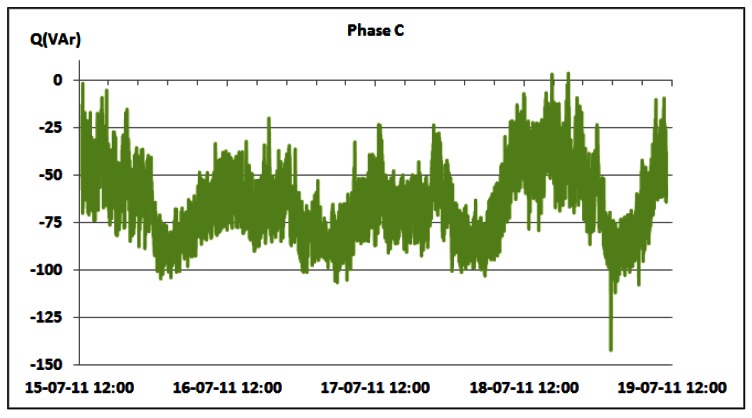
Reactive power of phase C for 15–19 July.

Changes in the reactive power consumption of the overall BS site for phases A, B and C are shown in Figures 18–20, respectively. Table 6 presents measured daily and total AC reactive power consumption for each of the phases. Compared with the measured values for the active power consumption (Tables 3 and 4), the reactive power consumption (Table 6) is approximately two orders of magnitude lower. Reactive power is caused by non-linear loads. Typical representatives in telecommunications are so-called switching power supplies used for powering equipment installed on the BS site. The switching power supply is an electronic power supply with an embedded switching regulator used for conversion of the electrical power. Similar to a conventional linear power supply, the switching supply transfers power from the electrical power grid to a load, which is typically some electronic device. Contrary to the linear power supply, the transistor of the switching power supply alternates rapidly from ON to OFF state. This approach results in minimal energy waste. However, switching currents generated by this type of supply cause electrical noise and, if not particularly treated, generally reduce the power factor. Hence, oscillations in the reactive power can be attributed to the operation of the mentioned electronic non-linear loads.

One exception is the case of phase A on which the air-conditioning system is connected with some other telecommunication equipment installed on site. Regulation circuitry of the air-conditioning system also contributes to the presence of the reactive power. Table 6 summarizes the aforementioned conclusions. We should point out that average values are omitted due to the nature of the reactive power. Negative values are attributed to the capacitive loads, and positive to the inductive loads. Averaging these values may result in erroneous conclusions.

**Figure 21.**
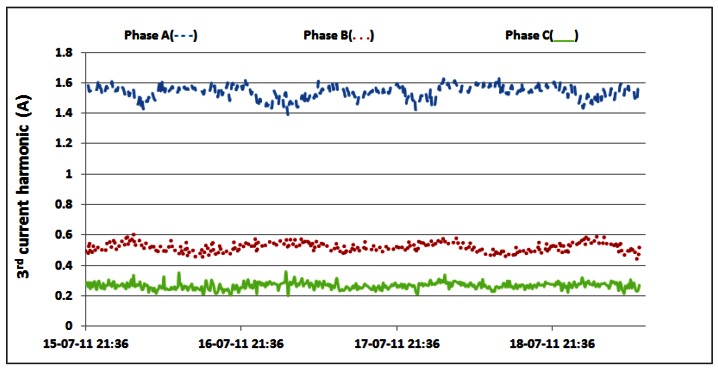
TRMS values of 3rd electric current harmonic measured on the individual phases.

**Figure 22.**
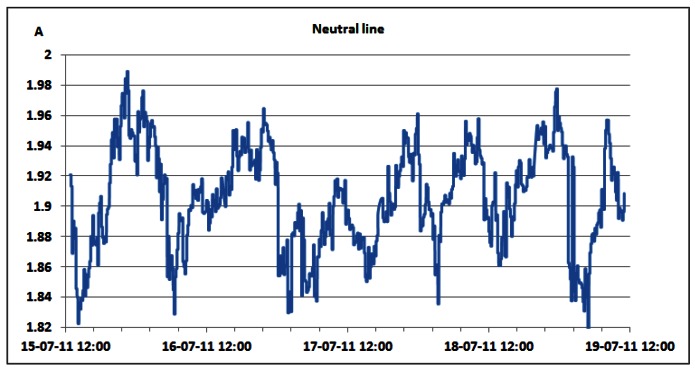
TRMS values of 3rd electric current harmonic measured on the neutral line.

**Figure 23.**
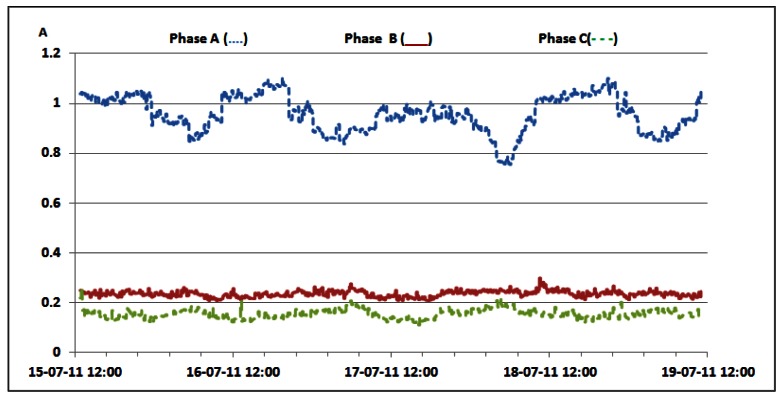
TRMS values of 5th electric current harmonic measured on the individual phases.

**Figure 24.**
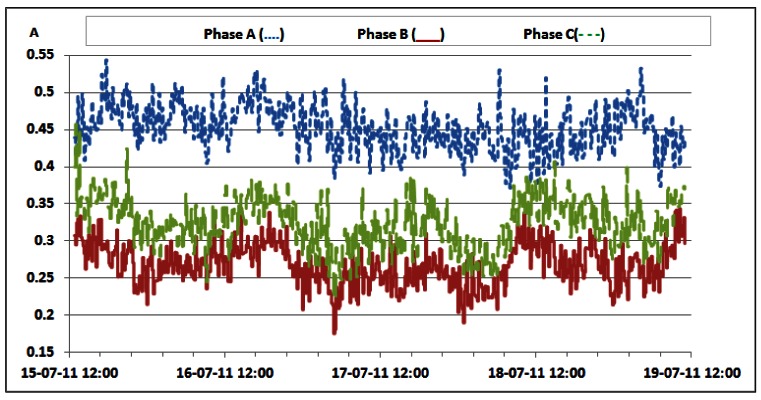
TRMS values of 7th electric current harmonic measured on the individual phases.

Additionally, Table 6 shows that oscillations between the minimum and maximum values for reactive powers are significantly lower than oscillations for active power (Tables 3 and 4). This result leads us to conclude that reactive power is quite constant and does not depend on the traffic variations. Since non-linear loads generate higher harmonics superimposed on the fundamental electric current, the influence of the non-linear loads on the reactive power can be verified through the presence of the odd current harmonics, as shown in Figures 21–24. It is worth to emphasize that higher harmonics in the electric current can be caused not only by non-linear loads, but also by higher harmonics in the voltage.

Nevertheless, in our case calculated total harmonic distortion for voltage (THDV) is equal to 1.85%, 1.84% and 1.82% for phases A, B and C, respectively. This proofs negligible influence of the voltage distortions on the higher current harmonics. Figure 21 shows TRMS measurements of the 3^rd^ current harmonic measured in the individual phases. Due to the nature of the three-phase supply system, contributions of the 3^rd^ current harmonics coming from the individual phases are summed in the neutral line, resulting in increased heating of the neutral line [22]. Because of that, it was reasonable to investigate the magnitude of the 3^rd^ current harmonic in the neutral line, as shown in Figure 22.

The neutral conductor of the supply system was additionally inspected and it was found to have a proper diameter to support the 3rd current harmonic. The presence of the 5th and 7th harmonic, measured in the individual phases, whose TRMS values are reported in Figures 23 and 24, further proves the existence of the non-linear loads [23]. Higher values in phase A are generated by the air-conditioning system. Nevertheless, it can be noticed that AC/DC converters and supporting circuitry provide stable filtering and DC conversion. This means that traffic variations do not have a significant influence on generation of the current harmonics, which results in their negligible influence on supply of the BS racks.

## 7. Power Consumption Modelling

Based on the measured average traffic load and the instantaneous power consumption obtained for each BS rack on the DC side, our goal was to develop a linear BS power consumption model. The developed model must express instantaneous power consumption of each BS rack as a function of the current traffic load. In order to model the interdependence between the power consumption of each BS rack and corresponding traffic load, we use the following equation:

(1) $$y=\beta_{1}f_{1}\left(x\right)+\ldots \beta_{p}f_{p}\left(x\right)+ɛ$$

According to Equation (1), response *y* is modelled as a linear combination of functions of independent variable *x* and a random error ε. In expression (1), *f_j_*(***x***) (*j* = 1, …, *p*) are the *terms* of the model, while *β_j_* (*j* = 1, …, *p*) represents the *coefficients*. It is assumed that the model has up to *p* different terms and corresponding coefficients. Uncontrolled factors and experimental errors are modelled in Equation (1) by *ε*, and assumed to be uncorrelated and distributed with zero mean and constant variance.

In our linear model, terms are *f*_1_(*x*) = 1 and *f*_2_(*x*) = *x*. For *n* independent observations (*x*_1_, *y*_1_), …, (*x_n_*, *y_n_*), the linear regression model becomes an *n × 2* system of equations:

(2) $$\left[\begin{array}{c} y_{1}\\ y_{2}\\ \vdots \\ y_{n} \end{array}\right]=\left[\begin{array}{cc} f_{1}\left(x\right) & f_{2}\left(x\right)\\ f_{1}\left(x\right) & f_{2}\left(x\right)\\ \vdots & \vdots \\ f_{1}\left(x\right) & f_{2}\left(x\right) \end{array}\right]\left[\begin{array}{c} \beta_{1}\\ \beta_{2} \end{array}\right]+\left[\begin{array}{c} {ɛ}_{1}\\ {ɛ}_{2}\\ \vdots \\ {ɛ}_{n} \end{array}\right]$$

or, in the matrix notation:

(3) y=Xβ+ɛ

The **X** in Equation (3) is the design matrix of the system (Jacobiana). To fit the model with the data, the system must be solved for the coefficient *β*_1_ and *β_2_*.

Ignoring the unknown errors **ε**, the matrix equation expressed in Equation (3) can be written and solved as follows:

(4) y=Xβ

(5) $${\text{X}}^{T}\text{y}={\text{X}}^{T}\text{X}\beta$$

(6) $$\beta ={\left({\text{X}}^{T}\text{X}\right)}^{-1}{\text{X}}^{T}\text{y}$$

For calculation of a confidence region, we need a variance *s* of coefficients *β_1_* and *β_2_*. The approximate variance-covariance matrix of the regression coefficients is estimated by [24]:

(7) $$s^{2}\left(\beta \right)=\frac{{ɛ}^{T}ɛ}{n-2}{\left({\text{X}}^{T}\text{X}\right)}^{-1}$$

The residual mean square has *n − p* degrees of freedom associated with it, since *p* parameters need to be estimated in the regression function for our model. The estimated standard uncertainty of the coefficient *β_1_* is on **s** (1,1), and for *β_2_* is on **s** (2,2) element of matrix **s**. For a normal distribution, the standard uncertainty covers the confidence level of approximately 68%. Multiplying the standard uncertainty by a coverage factor *k*, gives the result which is called the expanded uncertainty. Most commonly [25], uncertainty is scaled by using the coverage factor k = 2, in order to give a level of confidence equal to approximately 95%.

**Figure 25.**
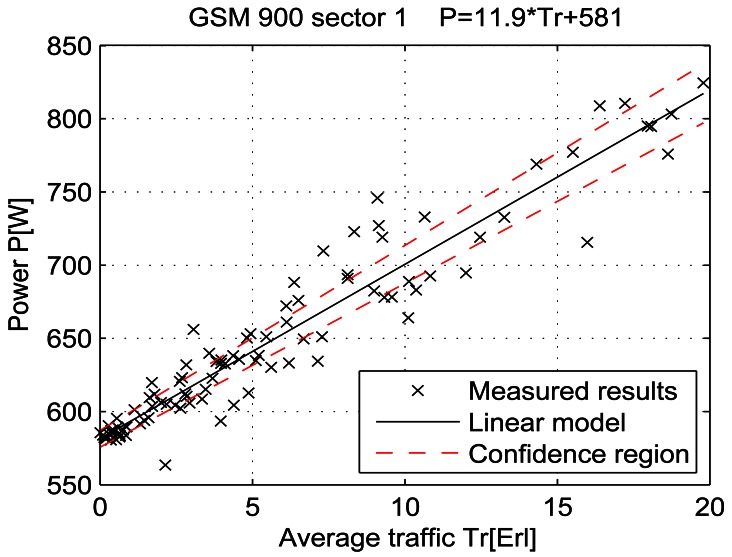
Power consumption model of GSM 900 sector 1 BS rack (total measuring period).

**Figure 26.**
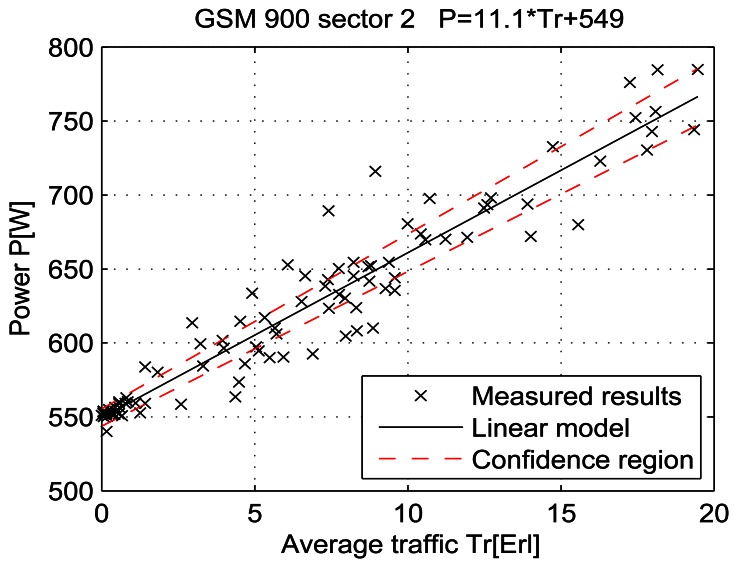
Power consumption model for GSM 900 sector 2 BS rack (total measuring period).

**Figure 27.**
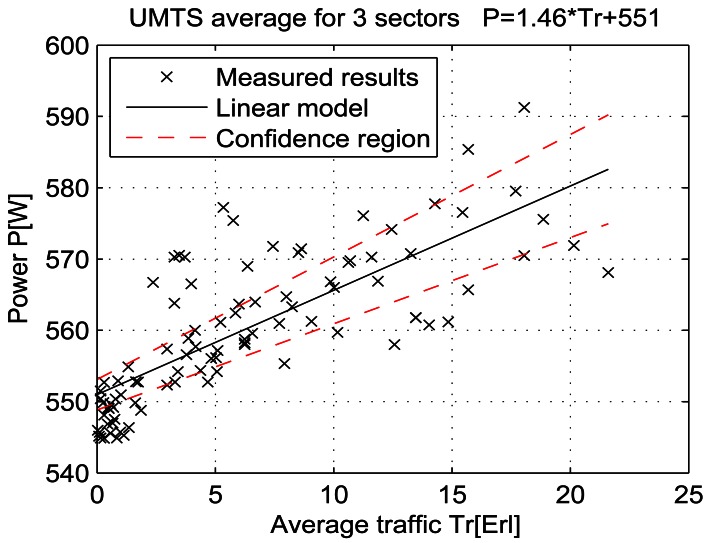
Power consumption model for UMTS BS rack (total measuring period).

**Figure 28.**
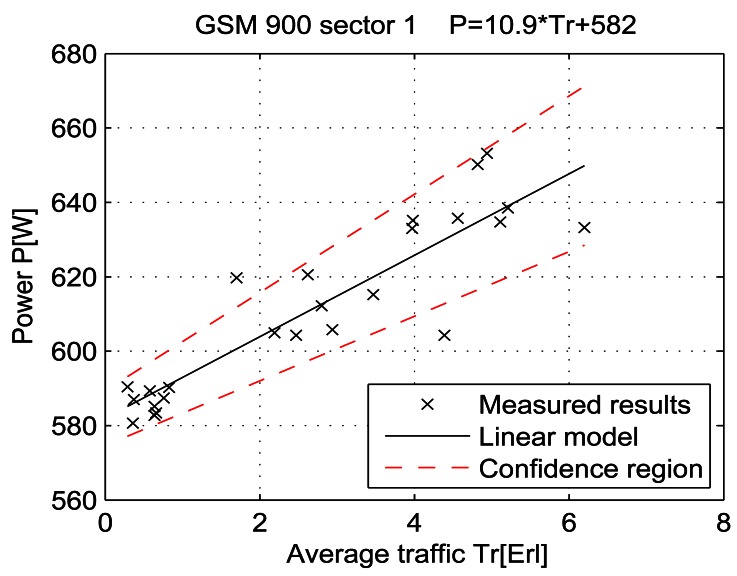
Power consumption model for GSM 900 sector 1 BS rack (Sunday 17/07).

**Figure 29.**
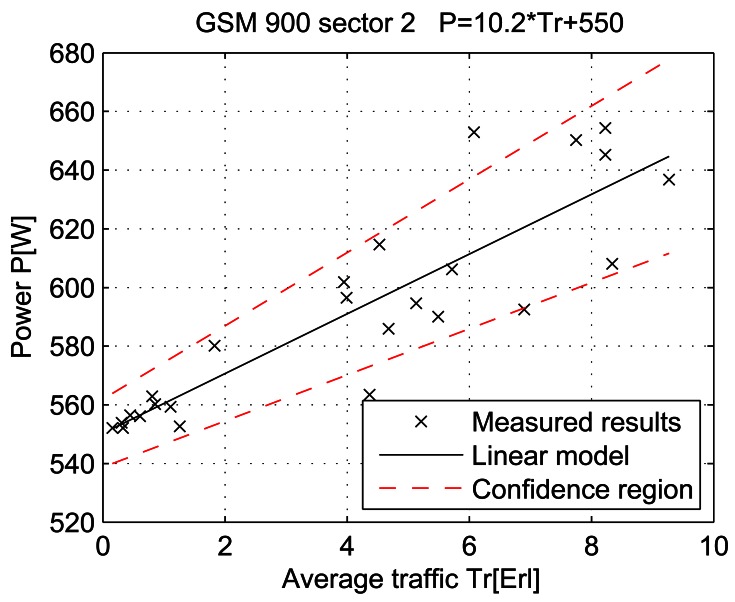
Power consumption model of GSM 900 sector 2 BS rack (Sunday, 17/07).

**Figure 30.**
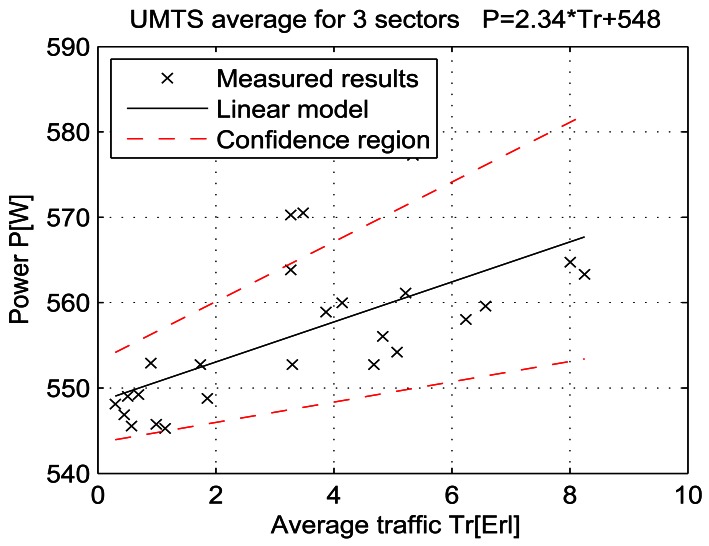
Power consumption model for UMTS BS rack (Sunday, 17/07).

**Figure 31.**
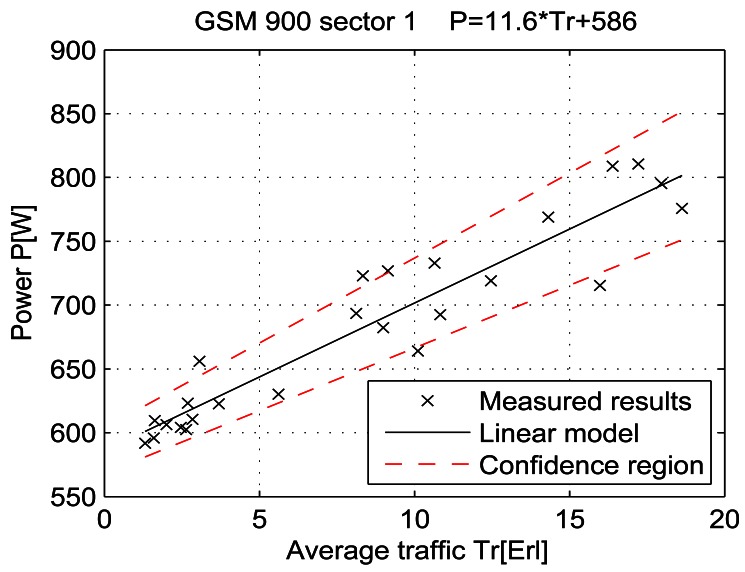
Power consumption model for GSM 900 sector 1 BS rack (Monday 18/07).

**Figure 32.**
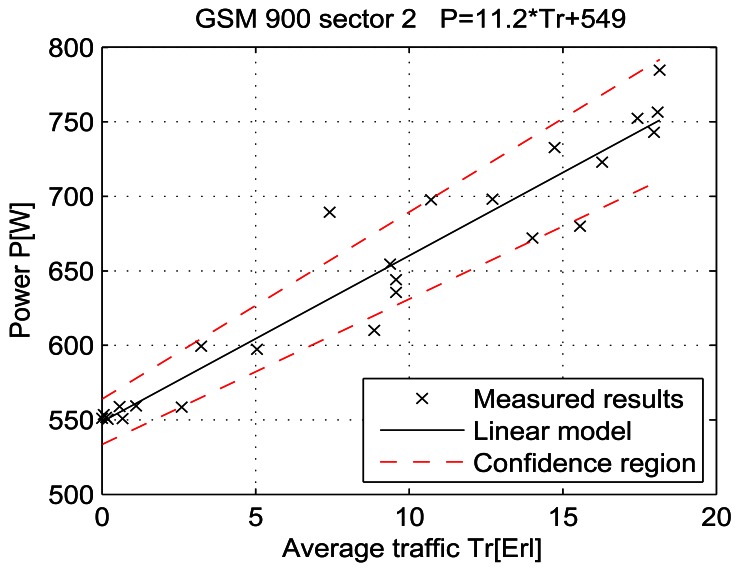
Power consumption model of GSM 900 sector 2 BS rack (Monday, 18/07).

**Figure 33.**
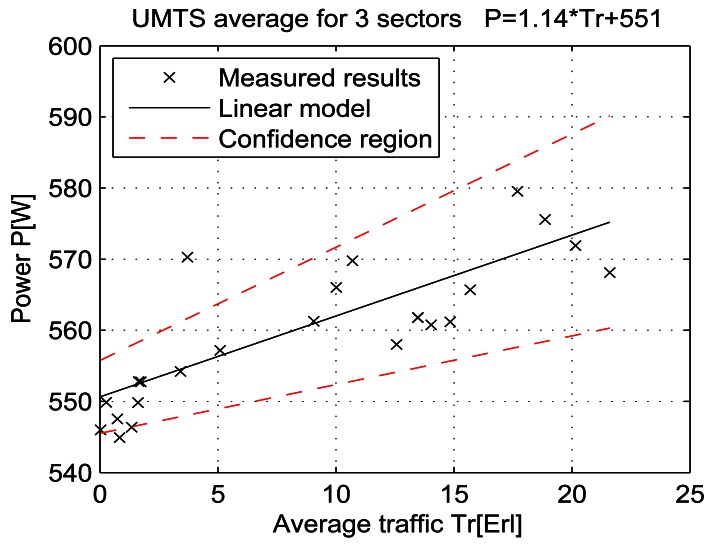
Power consumption model for UMTS BS rack (Monday, 18/07).

For a given period, from July 15 at 13:00 to July 19 at 11:00, let the telecommunication traffic Tr [Erl] be independent variable *x*, while the measured power P [W] is response *y*. Then the coefficients of the regression line are: *β_1_* [W], which represents the intercept, and *β_2_* [W/Erl] which represents the slope of the line. Calculations were performed by means of the function *regression* which is part of a Matlab software package. The obtained results are shown in Table 7 and in Figures 25–33. Table 7 presents the calculated values of coefficients *β_1_* and *β_2_* for the BS racks of different technologies. Calculated values have been shown for the cases of one working/weekend day and for the case of the total measuring period lasting five days. Additionally, for each of the analyzed cases, Table 7 lists mathematical expressions of the developed linear power consumption model.

In Figures 25–33, the developed linear models have been plotted together with the measured results and the linear regression lines having 95% confidence interval. According to these figures, we manage to model the linear dependence of the instantaneous power consumption on the traffic load. An increase in the traffic load results in a linear increase of the instantaneous BS power consumption and vice versa. Nevertheless, even when the traffic load is very low and can be neglected, the proposed linear models ensure some fixed amount of power consumption. Therefore, we propose linear models consisting of two components; fixed, which is not dependent on the traffic load, and variable, which is directly proportional to the traffic load. Due to previously presented differences in the traffic pattern between weekend and working days, some minor differences among these models can be noticed if we analyse Figures 28 and 31, 29 and 32, 30 and 33.

Generally, there is no major difference between models presenting power consumption of one day and the total power consumption for all five days of measurements. Each linear model corresponds to the specific BS technology and BSs of different technologies, manufacturers, production years or configurations might have different linear models. Since the proposed approach with a significant percentage of confidence pursues the results obtained through precise on-site measurements, the linear power consumption model can be accepted as a model for expressing the interdependence between instantaneous BS power consumption and traffic load. This cognition gives full confidence to usage of a linear model in future studies focused on improving the energy efficiency of already installed mobile radio access equipment.

## 8. Conclusions

In this paper, we provide an overview of the latest research activities focused on improving the energy efficiency of cellular access networks. Additionally, we perform an investigation regarding the impact of the traffic intensity on the power consumption of BSs. Analyses have been performed on a real indoor BS site containing BSs of GSM 900, GSM 1800 and UMTS access technologies. After five days of continuous measurements we obtained results which confirm that the instantaneous power consumption of BSs varies in accordance with the traffic load. This is a consequence of the direct correlation which exists between the BS electric current draw and the traffic load pattern.

This correlation is reflected in the active power consumption in each phase of the three-phase system used for electric supply of the complete macro BS site. Therefore, active site AC power consumption also varies according to changes in the traffic intensity. Additionally, we develop for each of the analyzed BSs a linear power consumption model. The proposed model with a significant percentage of confidence follows the results obtained through precise on-site measurements. Therefore, linear power consumption model can be accepted as a model for precise expression of the interdependence between instantaneous BS power consumption and traffic load. This interdependence is important for our future studies focused on improving the energy efficiency of already installed BSs.

## Figures and Tables

**Table 1.** Characteristics of base stations installed on analyzed site.

Characteristics of on-Site Base Stations	Base Station Model		

	GSM 900	GSM 1800	UMTS
Year of base station model production	2002	2009	2010
Frequency band	900 MHz	1,800 MHz	2,100 MHz
Number of base station racks	3	1	1
Number of transceivers in one base station rack	7-Sector 1		
	7-Sector 2	12	3
	4-Sector 3		
Number of sectors covered by one rack	1	3	3
Number of transceivers per sector	7/7/4	4/4/4	1/1/1
Number of antennas per sector	2	1	1
Number of combiners in rack	3	3	integrated in RF module
Number of antenna cables per antenna	2	2	2
Output power per sector	50 W	25 W	25 W
Antenna cable diameter	7/8″		
Average power consumption of site	7.5 kW		

**Table 2.** DC power consumption statistics.

Period	Consumption per base station	GSM 900 (Sector 1)	GSM 900 (Sector 2)	GSM 1800	UMTS
**Friday/15 July 2011**	Min. Daily power consumption [kW]	0.5567	0.5321	1.3751	0.5536
	Max. Daily power consumption [kW]	1.0125	0.9395	1.9115	0.6619
	Average daily power consumption [kW]	0.7064	0.6867	1.5820	0.5743
	Min./Aver. Power consumption ratio [%]	21.19	22.51	13.08	3.60
	Max./Aver. Power consumption ratio [%]	30.24	26.91	17.24	13.23
	Min./Max. Power consumption ratio [%]	45.02	43.36	28.07	16.36
	**Total energy consumption [kWh]**	**8.3760**	**8.1286**	**18.8147**	**6.8747**

**Saturday/16 July 2011**	Min. Daily power consumption [kW]	0.5545	0.5188	1.3450	0.5289
	Max. Daily power consumption [kW]	0.8435	0.7822	1.7175	0.6581
	Average daily power consumption [kW]	0.6282	0.6049	1.4569	0.5589
	Min./Aver. Power consumption ratio [%]	11.74	14.24	7.68	5.37
	Max./Aver. Power consumption ratio [%]	25.52	22.67	15.17	15.07
	Min./Max. Power consumption ratio [%]	34.26	33.68	21.69	19.63
	**Total energy consumption [kWh]**	**15.0385**	**14.4913**	**34.9149**	**13.4191**

**Sunday/17 July 2011**	Min. Daily power consumption [kW]	0.5509	0.5202	1.3415	0.5422
	Max. Daily power consumption [kW]	0.7560	0.7449	1.6819	0.6626
	Average daily power consumption [kW]	0.6282	0.6049	1.4569	0.5589
	Min./Aver. Power consumption ratio [%]	12.30	14.00	7.93	2.99
	Max./Aver. Power consumption ratio [%]	16.90	18.79	13.38	15.64
	Min./Max. power consumption ratio [%]	27.12	30.16	20.24	18.17
	**Total energy consumption [kWh]**	**14.6812**	**14.2074**	**34.4789**	**13.3499**

**Monday/18 July 2011**	Min. daily power consumption [kW]	0.5607	0.5176	1.3452	0.5419
	Max. daily power consumption [kW]	0.9892	0.9139	1.9739	0.6994
	Average daily power consumption [kW]	0.6883	0.6518	1.5453	0.5613
	Min./Aver. power consumption ratio [%]	18.54	20.59	12.95	3.46
	Max./Aver. power consumption ratio [%]	30.42	28.68	21.71	19.74
	Min./Max. power consumption ratio [%]	43.32	43.37	31.85	22.52
	**Total energy consumption [kWh]**	**16.4551**	**15.5838**	**36.9737**	**13.4769**

**Tuesday/19 July 2011**	Min. daily power consumption [kW]	0.5643	0.5512	1.3749	0.5417
	Max. daily power consumption [kW]	0.8653	0.8834	1.8429	0.6578
	Average daily power consumption [kW]	0.6090	0.5766	1.4202	0.5501
	Min./Aver. power consumption ratio [%]	7.33	4.41	3.19	1.53
	Max./Aver. power consumption ratio [%]	29.62	34.72	22.94	16.37
	Min./Max. power consumption ratio [%]	34.78	37.60	25.39	17.65
	**Total energy consumption [kWh]**	**6.6928**	**6.3675**	**15.3910**	**5.8308**

**Total energy consumption for all days [kWh]**		**52.9513**	**58.7786**	**140.5733**	**61.2436**

**Table 3.** Statistics of the AC daily active power consumption.

Period	Consumption per phase	Phase A	Phase B	Phase C
**Friday/15 July 2011**	Min. daily active power [kW]	3.02	2.06	2.03
	Max. daily active power [kW]	3.87	2.69	2.55
	Average daily active power [kW]	3.53	2.24	2.24
	Min./Aver. active power ratio [%]	14.35	8.04	9.39
	Aver./Max. active power ratio [%]	8.73	16.55	12.11
	Min./Max. active power ratio [%]	21.83	23.26	20.37
	**Total daily energy consumption [kWh]**	**91.54**		

**Saturday/16 July 2011**	Min. daily active power [kW]	2.73	2.03	1.99
	Max. daily active power [kW]	3.57	2.31	2.29
	Average daily active power [kW]	3.16	2.12	2.10
	Min./Aver. active power ratio [%]	13.91	4.44	5.04
	Aver./Max. active power ratio [%]	11.44	8.10	8.42
	Min./Max. active power ratio [%]	23.76	12.19	13.04
	**Total daily energy consumption [kWh]**	**175.07**		

**Sunday/17 July 2011**	Min. daily active power [kW]	2.66	1.97	2.00
	Max. daily active power [kW]	3.24	2.27	2.27
	Average daily active power [kW]	2.99	2.08	2.09
	Min./Aver. active power ratio [%]	11.07	5.06	3.98
	Aver./Max. active power ratio [%]	7.86	8.39	8.11
	Min./Max. active power ratio [%]	18.06	13.03	11.77
	**Total daily energy consumption [kWh]**	**169.18**		

**Monday/18 July 2011**	Min. daily active power [kW]	2.60	2.00	2.01
	Max. daily active power [kW]	3.79	2.55	2.55
	Average daily active power [kW]	3.23	2.18	2.19
	Min./Aver. active power ratio [%]	19.72	8.52	8.51
	Aver./Max. active power ratio [%]	14.80	14.36	13.93
	Min./Max. active power ratio [%]	31.60	21.65	21.26
	**Total daily energy consumption [kWh]**	**180.38**		

**Tuesday/19 July 2011**	Min. daily active power [kW]	2.48	1.38	1.41
	Max. daily active power [kW]	3.72	2.60	2.58
	Average daily active power [kW]	2.99	2.11	2.10
	Min./Aver. active power ratio [%]	17.22	34.83	32.77
	Aver./Max. active power ratio [%]	19.46	18.75	18.41
	Min./Max. active power ratio [%]	33.33	47.05	45.15
	**Total daily energy consumption [kWh]**	**79.88**		

**Table 4.** Total AC active power consumption statistics.

**Total period**	Min. daily active power [kW]	2.48	1.38	1.41
	Max. daily active power [kW]	3.87	2.69	2.58
	Average daily active power [kW]	3.16	2.14	2.14
	Min./Aver. active power ratio [%]	21.52	35.51	34.11
	Aver./Max. active power ratio [%]	18.35	20.45	17.05
	Min./Max. active power ratio [%]	35.92	48.70	45.35
	**Total energy [kWh]**	**604.51**		

**Table 5.** Estimation of consumed energy consumption and corresponding financial costs.

	LT (kWh)	HT (kWh)	Total (kWh)	Cost (€)
Working days	1,513.16	2,455.20	3,968.36	274.93
Saturdays	279.96	420.20	700.16	48.31
Sundays	276.88	402.20	679.08	46.77
Total/Month	2,070.00	3,277.60	5,347.60	370.01
Total/Year for analyzed BS site	24,840.00	39,331.20	64,171.20	4,440.12
Total for Operator/Year	29,808,000.00	47,197,440.00	77,005,440.00	5,328,144.00

**Table 6.** Daily and total AC reactive power consumption statistics.

Period	Consumption per phase	Phase A	Phase B	Phase C
**Friday/15 July 2011**	Min. daily reactive power [VAr]	−66.60	−78.10	−88.50
	Max. daily reactive power [VAr]	129.37	327.80	−3.00
**Saturday/16 July 2011**	Min. daily reactive power [VAr]	−75.40	−84.00	−104.60
	Max. daily reactive power [VAr]	88.78	−8.10	−20.20
**Sunday/17 July 2011**	Min. daily reactive power [VAr]	−84.10	−88.40	−106.60
	Max. daily reactive power [VAr]	43.58	−5.10	−23.40
**Monday/18 July 2011**	Min. daily reactive power [VAr]	−81.70	−79.00	−102.50
	Max. daily reactive power [VAr]	142.05	13.98	3.49
**Tuesday/19 July 2011**	Min. daily reactive power [VAr]	−77.70	−130.60	−141.80
	Max. daily reactive power [VAr]	123.21	21.799	−9.50
	**Min. daily reactive power [VAr]**	**142.05**	**327.80**	**0.00**
**Total period**	**Max. daily reactive power [VAr]**	−**84.10**	−**130.60**	−**14.80**

**Table 7.** Calculated regression coefficients with linear models for different BS technologies.

BS type	Period	*β_1_* ± 2s (1,1)	*β_2_* ± 2s (2,2)	Total measuring period	Sunday 17/07	Monday 18/07
		[W]	[W/Erl]			
**GSM 900 sector 2**	Total measuring period	549 ± 5.8	11.1 ± 0.68	P = 11.1 * Tr + 549	P = 10.2 * Tr + 550	P = 11.2 * Tr + 549
	Sunday 17/07	550 ± 11.6	10.2 ± 2.32			
	Monday 18/07	549 ± 15.1	11.2 ± 1.40			
**GSM 900 sector 1**	Total measuring period	581 ± 5.6	11.9 ± 0.73	P = 11.9 * Tr + 581	P = 10.9 * Tr + 582	P = 11.6 * Tr + 586
	Sunday 17/07	582 ± 7.3	10.9 ± 2.26			
	Monday 18/07	586 ± 17.7	11.6 ± 1.75			
**UMTS**	Total measuring period	550 ± 2.1	1.46 ± 0.25	P = 1.46 * Tr + 551	P = 2.34 * Tr + 548	P = 1.14 * Tr + 551
	Sunday 17/07	548 ± 4.7	2.34 ± 1.15			
	Monday 18/07	551 ± 5.1	1.14 ± 0.45			

## References

[1] Humar I., Ge X., Xiang L., Jo M., Chen M., Zheng J. (2011). Rethinking energy efficiency models of cellular networks with embodied energy. IEEE Netw..

[2] Deruyck M., Vereecken W., Tanghe E., Joseph W., Pickavet M., Martens L., Demeester P. Power consumption in wireless access networks.

[3] Blume O., Eckhardt H., Klein S., Kuehn E., Wajda W.M. (2010). Energy savings in mobile networks based on adaptation to traffic statistics. Bell Labs Tech. J.

[4] Oh E., Krishnamachari B., Liu X., Niu Z. (2011). Toward dynamic energy-efficient operation of cellular network infrastructure. IEEE Commun. Mag..

[5] Richter F., Fehske A.J., Fettwe G.P. Energy efficiency aspects of base station deployment strategies for cellular networks.

[6] Auer G., Giannini V., Desset C., Godor I., Skillermark P., Olsson M., Imran M.A., Sabella D., Gonzalez M.J., Blume O., Fehske A. (2011). How much energy is needed to run a wireless network?. IEEE Wirel. Commun..

[7] Chen T., Haesik H., Yang Y. Energy efficiency metrics for green wireless communications.

[8] Correia L.M., Zeller D., Blume O., Ferling D., Jading Y., Godor I., Auer G., van der Perre L. (2010). Challanges and enabling technologies for energy aware mobile radio networks. IEEE Commun. Mag..

[9] Forster C., Dickie I., Maile G., Smith H., Crisp M. Understanding the Enviromental Impact of Communication Systems: Ofcom Study Report.

[10] Hirata H., Totani K., Maehata T., Shimura T., Take M., Jurokawa Y., Onishi M., Ada Y., Hirata Y. (2010). Development of high efficiency amplifier for cellular base stations. SEI Tech. Rev..

[11] Zoican S. (2008). The role of programmable digital signal processors (dsp) for 3 g mobile communication systems. ACTA Tech. Napoc..

[12] Roy S.N. Energy logic: A road map to reducing energy consumption in telecommunications networks.

[13] Etoh M., Ohya T., Nakayama T. Energy consumption issues on mobile network systems.

[14] Automatic Terminal Information Service (ATIS) Report on Wireless Network Energy Efficiency.

[15] Conte A., Feki A., Chiaraviglio L., Ciullo D., Meo M., Marsan M.A. (2011). Cell wilting and blossoming for energy efficiency. IEEE Wirel. Commun..

[16] Han C., Harrold T., Armour S., Krikidis I., Videv S., Grant P.M., Haas H., Thompson J.S., Ku I., Wang C.X. (2011). Green radio: Radio techniques to enable energy-efficient wireless networks. IEEE Commun. Mag..

[17] Tripper D., Rezgui A., Krishnamurthy P., Pacharintankul P. Dimming cellular networks.

[18] Niu Z., Wu Y., Gong J., Yang Z. (2010). Cell zooming for cost-efficient green cellular networks. IEEE Commun. Mag..

[19] Niu Z. (2011). TANGO: Traffic-aware network planning and green operation. IEEE Wirel. Commun..

[20] Marsan M.A., Meo M. (2010). Energy efficient management of two cellular access networks. ACM SIGMETRICS Perform. Eval. Rev..

[21] Chen T. (2011). Network energy saving technologies for green wireless access networks. IEEE Wirel. Commun..

[22] Željko N. (2006). Handbook on Power Quality.

[23] (2006). Power System Network Regulations.

[24] Kutner M. (2004). Applied Linear Regression Models.

[25] Joint Committee: Evaluation of Measurement Data—An Introduction to the Guide to the Expression of Uncertainty in Measurement. http://www.bipm.org.

